# Age and sex disparities in drug shortage impacts: a 10-year nationwide study in France

**DOI:** 10.1093/eurpub/ckag045

**Published:** 2026-03-28

**Authors:** Laëtitia Belgodère, Christopher Leleu, Trystan Bacon, Pascale Daynes, Christophe Decoene, Patrick Feugier, Roseline Mazet, Thierry Vial, Stéphane Vignot, Mehdi Benkebil, Wahiba Oualikene-Gonin, Patrick Maison

**Affiliations:** Agence Nationale de Sécurité du Médicament et des Produits de Santé (ANSM), Saint Denis, France; Agence Nationale de Sécurité du Médicament et des Produits de Santé (ANSM), Saint Denis, France; Agence Nationale de Sécurité du Médicament et des Produits de Santé (ANSM), Saint Denis, France; Collège universitaire de médecine générale, Université Claude Bernard Lyon1, Lyon, France; Agence Nationale de Sécurité du Médicament et des Produits de Santé (ANSM), Saint Denis, France; Agence Nationale de Sécurité du Médicament et des Produits de Santé (ANSM), Saint Denis, France; Pôle anesthésie-réanimation, Centre hospitalier universitaire de Lille, Lille, France; Agence Nationale de Sécurité du Médicament et des Produits de Santé (ANSM), Saint Denis, France; Service de Chirurgie Vasculaire et Endovasculaire (LYVES), Université Claude Bernard Lyon 1, Lyon, France; Agence Nationale de Sécurité du Médicament et des Produits de Santé (ANSM), Saint Denis, France; Centre hospitalier universitaire de Grenoble-Alpes, Grenoble, France; Agence Nationale de Sécurité du Médicament et des Produits de Santé (ANSM), Saint Denis, France; Hospices Civils de Lyon, Lyon, France; Agence Nationale de Sécurité du Médicament et des Produits de Santé (ANSM), Saint Denis, France; EA7509 Université Reims Champagne Ardenne, Reims, France; Institut Godinot, Reims, France; Agence Nationale de Sécurité du Médicament et des Produits de Santé (ANSM), Saint Denis, France; Agence Nationale de Sécurité du Médicament et des Produits de Santé (ANSM), Saint Denis, France; Agence Nationale de Sécurité du Médicament et des Produits de Santé (ANSM), Saint Denis, France; CHI Créteil, Créteil, France; EA 7379, Epiderme, Faculté de Santé, Université Paris-Est Créteil, Créteil, France

## Abstract

Drug shortages are a growing public health issue, unevenly impacting therapeutic classes. Despite variations in drug consumption across populations, the consequences of these shortages on different patient groups remain insufficiently characterized. This study investigated age and sex profiles of patients consuming the therapeutic classes most commonly affected by drug shortages in France. The age and sex risk of shortage exposure were estimated in a nationwide retrospective study of French patients between 2014 and 2023, using data from the French drug shortage notification system and the national health insurance for the entire French population. Over 10 years, 17 505 drug shortage reports were recorded, 60.8% involving cardiovascular, nervous system, and anti-infective agents. Significantly higher mean percentages of consumers for drugs concerned by shortage reports per 1000 were observed in ≥60-year-old patients for 12 of the 14 therapeutic classes, compared to 20–59-year-old patients (*P* = .002). Younger population was more significantly concerned by anti-infective agents (11.2 vs 9.2, *P* = .002), respiratory system (1.8 vs 1.5, *P* = .002), and sensory organ (0.7 vs 0.6, *P* = .002) classes. Significantly higher mean percentages of consumers for drugs concerned by shortage reports per 1000 were observed for all Anatomical Therapeutic Chemical 1st level classes in women (*P* = .002), but agents acting on the renin–angiotensin system were higher for men (2.32 vs 2.02; *P* = .002). Our exploratory results suggest that a population-level approach is essential to understand how shortages affect different groups and exacerbate health inequalities.

## Introduction

In many countries, drug shortages have emerged as an escalating public health concern in recent years, with profound implications for patients care [1–3]. Reports of drug shortages, largely driven by disruptions in pharmaceutical manufacturing and supply chains, have surged over the past decade, reflecting a troubling trend that poses challenges to healthcare systems worldwide [1, 4, 5]. The growing media coverage of this issue exacerbates patient anxiety, regardless of their direct encounter with drug shortages, creating a climate of uncertainty around drug availability. In France, the National Agency for the Safety of Medicines and Health Products (ANSM) centralizes reports and coordinates efforts to mitigate the impact of limited drug availability on healthcare delivery [6].

Since 2012 [7], France has progressively strengthened its regulatory framework to address drug shortages. The focus has been on ensuring the availability of major therapeutic interest (MTI) drug for which interrupting treatment is likely to jeopardize the patient’s vital prognosis, or represents a significant loss of chance in view of the seriousness or progressive potential of the disease. Key measures include the 2016 decree [8] mandating manufacturers to promptly notify the ANSM of any potential or actual disruption in the MTI drugs’ supply chain that could lead to a shortage or face financial penalties, followed by the decree in 2021 [9] requiring marketing authorization holders (MAH) to maintain a minimum safety stock. In addition, MAHs are required to submit a shortage management plan, which identifies supply chain risks and outlines measures to mitigate them.

The causes of drug shortages are multifaceted [6], encompassing issues such as production delays, regulatory challenges, and market dynamics. While certain studies [6, 10–14] have examined the underlying causes of drug shortages, data on their impact on patients remain limited [15]. Some research has highlighted risks [16–20] such as adverse drug reactions, reduced therapeutic efficacy, and medication errors that can occur when substitutions are required, yet there is a lack of comprehensive data on how these shortages impact patients directly. Some questions therefore remain unanswered, such as the clinical consequences for the patient, information-related stress or the issue of who is most impacted. An overview of the causes is necessary to prevent shortages, but a description of the population is crucial to prevent the consequences. Previous studies suggest that drug shortages affect Anatomical Therapeutic Chemical (ATC) classes unevenly, with certain therapeutic categories more affected than others [6, 13, 21]. As drug consumption varies across populations, these shortages create varying degrees of vulnerability among patient groups. A population-based approach is therefore essential to address this public health issue.

The aim of the study was to investigate the age and sex profiles of patients affected by consumption of the therapeutic classes most commonly impacted by drug shortages in France over the past decade.

## Methods

### Study design

We performed a nationwide retrospective study based on secondary analysis of two national administrative databases concerning drug shortage reports and drug reimbursements by the national health insurance for the entire French population between 2014 and 2023.

### Data sources

#### Data from the French drug shortage notification system

MAH and pharmaceutical companies exploiting MTI drugs are required to promptly notify the ANSM of any actual disruption to the supply chain, termed a shortage, or potential disruption likely to result in unavailability of the drug, termed a shortage risk. Our observational study analysed all MTI drug shortage reports from the French national system from 1 January 2014 to 31 December 2023. Variables included report type (shortage or shortage risk), ATC code and presentation identifier code (CIP) (registered only from May 2021).

#### Data from the French national health data system

Pharmacy drug reimbursement data, between 1 January 2014 and 31 December 2023, were extracted and aggregated from the exhaustive French National Health Data System (SNDS), the national health insurance database covering over 99% of the French population, via the open-access Open Medic [22] database. The database contains anonymized information about reimbursed drugs, together with sociodemographic data (e.g. ATC codes, CIP, number of consumers and number of reimbursed packages by age group and sex). Datasets, compiled yearly, provide consumer counts at different grouping levels (CIP, ATC1 and ATC2), categorized by age group (<20, 20–59, ≥60 years) and sex (woman or man). The number of consumers refers to patients with at least one reimbursement in the ATC class during the year. Drugs with unknown codes were excluded.

#### Demographic data

Population estimates for each year between 2014 and 2023 were obtained from the French National Institute of Statistics and Economic Studies (INSEE) [23] which is the national body responsible for official statistical data, including population accounting with demographic indicators. INSEE provides annual population estimates based on rolling censuses and administrative records, ensuring comprehensive coverage of France’s resident population on January 1 each year. These estimates cover the entire population and are considered exhaustive for national and sub-national statistical purposes. Data are classified by age group (<20, 20–59, ≥60 years) and sex, providing annual population counts for each subgroup. Data from the 16 January 2024 were used.

### Analysis plan

Data from the three sources were cross-referenced by year using ATC class (ATC1 or ATC2 level) or CIP. The resulting datasets were stratified by population groups (age or sex). Analyses were conducted on an annual basis over the 10-year period and performed using XLSTAT software (version 2023.3.0.1415, Addinsoft, Paris, France). The ATC classification system (according to the World Health Organization) [24] is an international standard for categorizing drugs by target organ or system and therapeutic, pharmacological and chemical properties. Drugs are classified in groups at a five-level hierarchy. This study focuses on these first two levels, with the first level (ATC1) consisting of 14 main anatomical groups and the second level (ATC2) consisting of 99 pharmacological subgroups.

A descriptive analysis of the shortage report data was performed annually over the 10-year period. Dichotomous variables were described using percentages and numbers. The total number of reports was recorded and categorized by type. Trends were analysed overall and by ATC classes. The frequency of being reported in shortage within the year (total number of reports/total number of CIP drugs reimbursed), and probabilities of the report impacting the ATC class (number of reports in ATC class/total number of reports) were calculated.

Annual drug consumption over the 10-year study period was analysed, stratified by age group and sex. Total numbers of drug consumptions and reimbursed drug packages were calculated, and distributions were expressed as percentages.

Demographic trends in the French population were assessed from 2014 to 2023. Annual proportions of women, <20-year-olds, and ≥60-year-olds were calculated.

Annual drug consumption was analysed using reimbursement claims and population size. For each ATC class and year, two population-level measures of consumption were computed: the proportion of consumers among the French population (patients with ≥1 reimbursement, reflecting use prevalence) and the consumption rate (mean reimbursed drug packages per capita, reflecting volume). These measures, referred to as the annual probability of consuming this ATC class, were analysed by population. Continuous variables were summarized as 10-year means ± standard deviations.

A consumption indicator for drugs concerned by shortage reports was defined by ATC class and population group as follows: (frequency of reported shortages in the year) × (frequency of the ATC class among reported shortages in the year) × (annual probability of consuming this ATC class in the population). For each ATC1 class, 10-year mean percentage of consumers and mean consumption rates for drugs concerned by shortage reports were calculated and compared using the two-tailed Wilcoxon signed-rank test. Comparisons were made using 20–59-year-olds as reference (<20 vs. 20–59, and ≥60 vs. 20–59), and women vs. men. Tied values were handled using the Hollander and Wolfe method. Significance thresholds were *P* < .00357 according to the Bonferroni correction for multiple comparisons with the 14 ATC1 classes. ATC2 analyses were restricted to selected classes to limit multiple comparisons. For consumers of therapeutic classes affected by shortages, ATC2 classes representing >50% of reported shortages over 10 years were included with age- and sex-stratified comparisons (*P*-adjusted < .005). For the consumption rate of drugs concerned by shortage reports, ATC1 classes showing discrepancies between prevalence of use and consumption volume were first selected, and ATC2-level tests were performed within these classes. In ≥60-year-olds, analyses focused on class G (4 sub-classes, *P*-adjusted < .0125), while in <20-year-olds classes J, R, and S (14 sub-classes, *P*-adjusted < .00357) were considered. Sex-specific analyses targeted classes B and C (14 sub-classes, *P*-adjusted < .00357).

## Results

### Reports of a shortage in the availability of MTI drugs in France

In 2023, 3 356 drug specialities out of 12 625 were reported to be in shortage or at shortage risk at least once during the year (26.6%). Of these reports, 3 370 concerned shortage risks, representing 68.4% of all reports.

**Figure 1.**
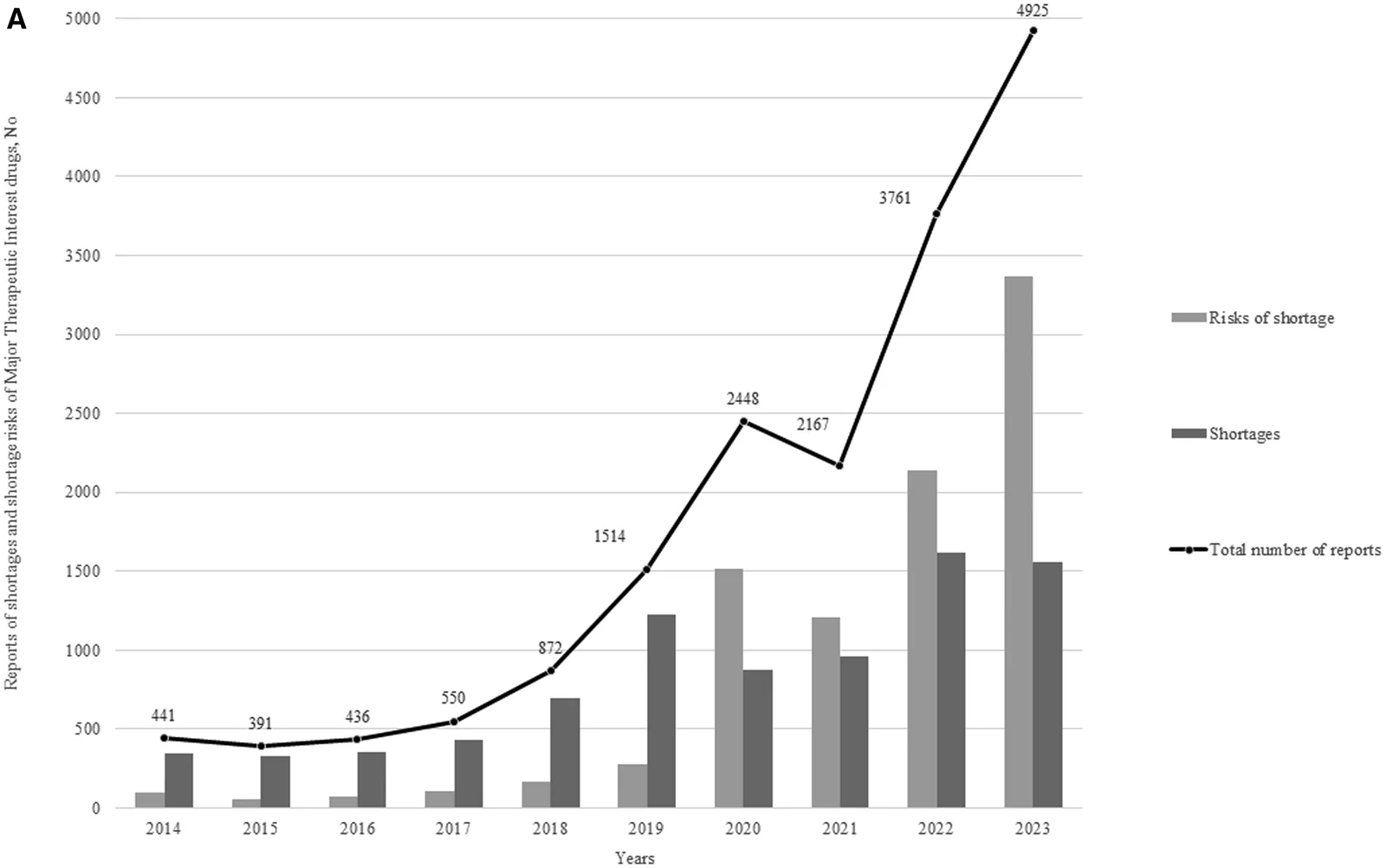
Number of reports of shortage risks and shortages for major therapeutic interest drugs between 2014 and 2023 in France.

A total of 17 505 reports were reported in France between the 1 January 2014 and the 31 December 2023. Figure 1 shows the trend in the total number of reports over this period (from 441 to 4 925), and details the distribution by type of report.

**Figure 2.**
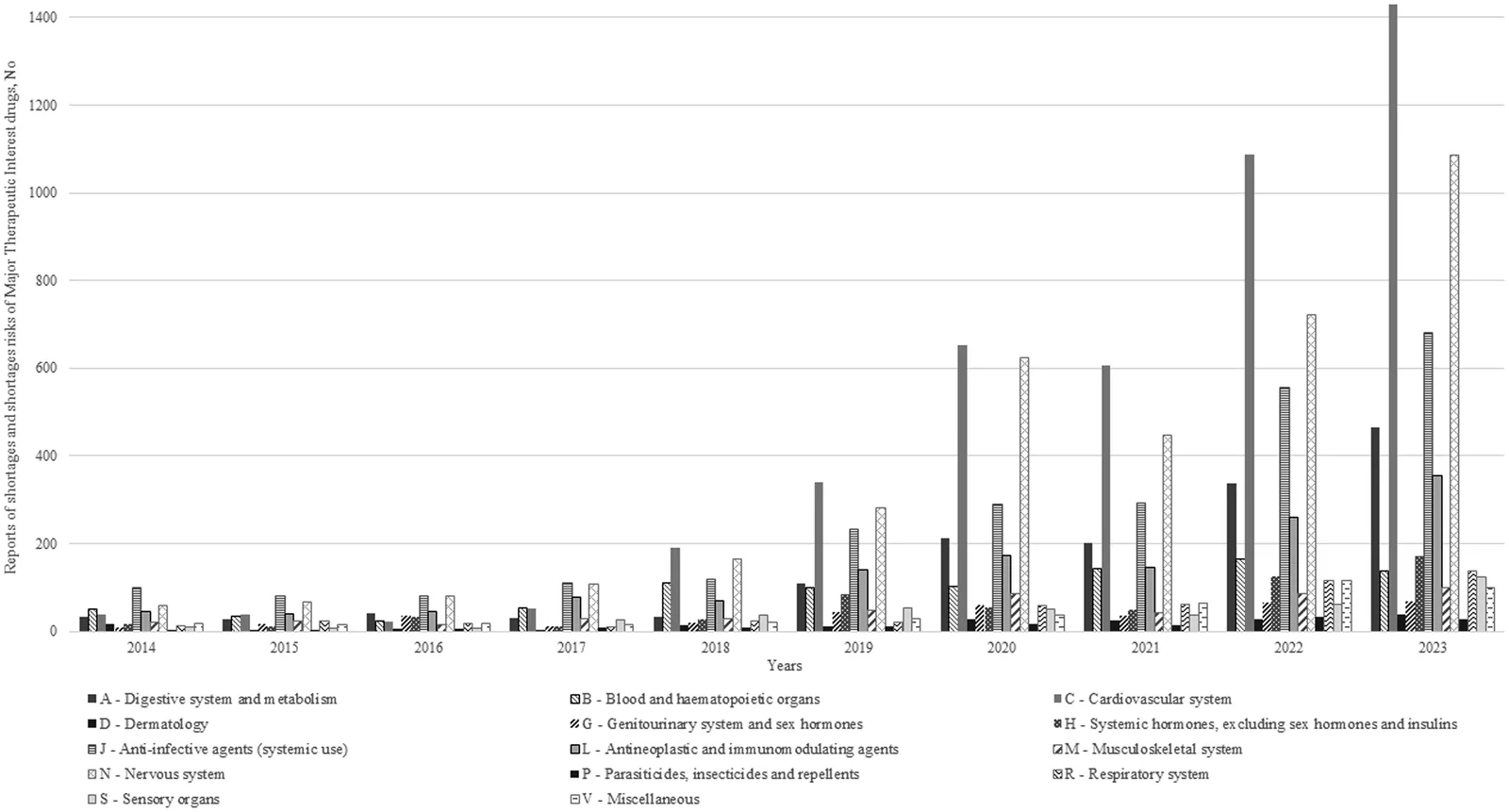
Number of reports of shortage risks and shortages for major therapeutic interest drugs between 2014 and 2023 in France according to therapeutic class.

Figure 2 shows that therapeutic classes were reported differently in shortage reports during the period. The ATC1 classes that were most highly affected over time were the cardiovascular system, the nervous system and anti-infective agents, accounting for 60.8% of reports (*n* = 10 639).

Ten ATC2 classes accounting for more than half of the drug shortage reports during the period: agents acting on the renin–angiotensin system (10.8%), antibacterials for systemic use (8.5%), psycholeptics (5.0%), antineoplastic agents (5.0%), lipid-modifying agents (4.3%), analgesics (4.1%), psychoanaleptics (3.8%), antiepileptics (3.6%), beta-blocking agents (3.3%) and drugs used in diabetes (3.1%).

### Population characteristics from 2014 to 2023

The French population increased from 66 130 873 subjects in 2014 to 68 143 433 subjects in 2023, with a stable sex ratio (51.6% women). The ≥60-year-old population increased from 24.2% to 27.4%, while the <20-year-old population decreased from 24.6% to 23.5%.

### Drug consumption from 2014 to 2023

During the study period, 6 601 005 999 drug consumptions were recorded, with a mean annual consumption of 660 100 600 (±27 054 131). Women represented the majority of consumers (58.4% vs. 41.6% male). By age, 20–59-year-olds constituted the largest group (43.6%), followed by ≥60-year-olds (40.7%) and <20-year-olds (15.6%). Supplementary Table S1 provides proportions of individuals in each age group or sex consuming at least one drug in the ATC1 class, adjusted for population size and stratified by therapeutic class.

When the quantities of drugs are taken into account, women also remained the main consumers, accounting for 58.0% of total reimbursed packages, compared with 42.0% for men. By age, ≥60-year-olds consumed the largest quantity of drug packages (54.8%), followed by 20–59-year-olds (37.2%) and <20-year-olds (7.9%). These overall patterns of drug package consumption by sex and age are detailed in Supplementary Table S2, which presents the mean annual per capita consumption rates across different therapeutic classes.

### Consumers of therapeutic classes concerned by shortage reports over the 10-year study

For most therapeutic classes concerned by shortage reports, the annual number of consumers per 1000 was higher with increasing age (Table 1). In ≥60-year-olds, results were significantly higher than in 20–59-year-olds for all classes, except for G (Genitourinary system and sex hormones) and P (Parasiticides, insecticides and repellents), for which they were lower. In <20-year-olds, results were significantly higher than in 20–59-year-olds for classes J (anti-infective), R (respiratory system), and S (sensory organs).

**Table 1.** Comparison of 10-year mean number of consumers per 1000 capita for therapeutic classes concerned by shortage reports, according to age group by therapeutic class (ATC) first level (Wilcoxon signed-rank test)

ATC 1 class		Number of consumers per 1000 capita for therapeutic classes concerned by shortage reports				
		20–59 years	<20 years	<20 vs 20–59 years	≥60 years	≥60 vs 20–59 years
		Mean (SD)	Mean (SD)	*P*-value	Mean (SD)	*P*-value
A	Digestive system and metabolism	6.4 (6.6)	5.9 (6.1)	.002	9.4 (9.7)	.002
B	Blood and haematopoietic organs	1.1 (0.6)	0.4 (0.2)	.002	3.0 (1.6)	.002
C	Cardiovascular system	4.9 (5.4)	0.3 (0.4)	.002	23.2 (25.5)	.002
D	Dermatology	0.5 (0.3)	0.4 (0.3)	.019	0.6 (0.4)	.002
G	Genitourinary system and sex hormones	0.6 (0.3)	0.2 (0.1)	.002	0.5 (0.3)	.002
H	Systemic hormones, excluding sex hormones and insulins	1.1 (1.0)	1.0 (0.9)	.002	1.5 (1.4)	.002
J	Anti-infective agents (systemic use)	9.2 (7.5)	11.2 (9.1)	.002	14.0 (11.6)	.002
L	Antineoplastic and immunomodulating agents	0.2 (0.2)	0.0 (0.0)	.002	0.5 (0.5)	.002
M	Musculoskeletal system	1.6 (0.9)	0.9 (0.4)	.002	1.7 (1.0)	.002
N	Nervous system	19.5 (18.8)	19.1 (18.4)	1.000	22.3 (21.2)	.002
P	Parasiticides, insecticides and repellents	0.04 (0.03)	0.04 (0.03)	.492	0.03 (0.02)	.002
R	Respiratory system	1.5 (1.4)	1.8 (1.7)	.002	1.6 (1.4)	.002
S	Sensory organs	0.6 (0.5)	0.7 (0.6)	.002	1.1 (0.9)	.002
V	Miscellaneous	0.3 (0.2)	0.1 (0.0)	.002	0.5 (0.5)	.002

Annual mean number of consumers per 1000 was higher in women for all affected therapeutic classes (*P* = .002, Table 2).

**Table 2.** Comparison of 10-year mean number of consumers per 1000 capita for therapeutic classes concerned by shortage reports, according to sex by therapeutic class (ATC) 1st level (Wilcoxon signed-rank Test)

ATC 1 class		Number of consumers per 1000 capita for therapeutic classes concerned by shortage reports		
		Men	Women	
		Mean (SD)	Mean (SD)	*P*-value
A	Digestive system and metabolism	6.2 (6.4)	7.9 (8.2)	.002
B	Blood and haematopoietic organs	1.3 (0.7)	1.6 (0.9)	.002
C	Cardiovascular system	8.5 (9.6)	8.8 (9.8)	.002
D	Dermatology	0.4 (0.3)	0.5 (0.3)	.002
G	Genitourinary system and sex hormones	0.2 (0.1)	0.7 (0.4)	.002
H	Systemic hormones, excluding sex hormones and insulins	0.9 (0.9)	1.4 (1.3)	.002
J	Anti-infective agents (systemic use)	10.0 (8.2)	11.9 (9.7)	.002
L	Antineoplastic and immunomodulating agents	0.2 (0.2)	0.3 (0.3)	.002
M	Musculoskeletal system	1.3 (0.7)	1.6 (0.9)	.002
N	Nervous system	18.7 (18.0)	21.5 (20.6)	.002
P	Parasiticides, insecticides and repellents	0.03 (0.02)	0.05 (0.03)	.002
R	Respiratory system	1.5 (1.3)	1.7 (1.6)	.002
S	Sensory organs	0.6 (0.6)	0.8 (0.7)	.002
V	Miscellaneous	0.3 (0.2)	0.3 (0.3)	.002

The same trend was observed among the 10 studied ATC2 classes, which accounted for more than half of all shortage reports over the 10-year period.

Higher results among ≥60-year-olds were observed for six ATC2 classes: Agents acting on the renin–angiotensin system (7.13 ± 7.87 vs 1.23 ± 1.35; *P* = .002), Antibacterials for systemic use (5.88 ± 6.72 vs 5.48 ± 6.26; *P* = .002), analgesics (4.62 ± 5.86 vs 4.24 ± 5.46; *P* = .002), tantiepileptics (0.27 ± 0.19 vs 0.14 ± 0.10; *P* = .002), psycholeptics (2.19 ±2.47 vs 1.29 ± 1.47; *P* = .002), and psychoanaleptics (0.92 ± 1.01 vs 0.54 ± 0.60; *P* = .002).

By sex, mean number of consumers per 1000 for therapeutic classes concerned by shortage were significantly higher in women in 5 of the 10 studied ATC2 classes: antibacterials for systemic use (5.17 ± 4.73 vs 4.19 ± 3.83; *P* = .002), analgesics (3.88 ± 4.58 vs 3.36 ± 3.99; *P* = .002), antiepileptics (0.14 ± 0.10 vs 0.12 ± 0.08; *P* = .002), psycholeptics (1.35 ±1.46 vs 0.87 ± 0.95; *P* = .002), and psychoanaleptics (0.61 ± 0.67 vs 0.33 ± 0.36; *P* = .002). Number of consumers per 1000 for therapeutic classes concerned by shortage was significantly higher in men only for agents acting on the renin–angiotensin system (2.32 ± 2.31 vs 2.02 ± 2.00; *P* = .002).

### Consumption of drugs concerned by shortage reports over the 10-year study

When accounting for the number of reimbursed packages, an additional therapeutic class emerged for ≥60-year-olds, the genitourinary system (4.5 ± 2.6 vs 2.8 ± 1.5; *P* = .002), primarily driven at the ATC2 level by urologicals (0.39 ± 0.38 vs 0.03 ± 0.03; *P* = .008). Using the same approach, results for <20-year-olds regarding anti-infective agents were confirmed, with higher 10-year mean consumption rates per 1000 for drugs concerned by shortage reports compared to 20–59-year-olds (42.7 ± 35.7 vs 36.8 ± 28.9; *P* = .002), driven at the ATC2 level by vaccines in class J (1.22 ± 0.66 vs 0.19 ± 0.10; *P* = .002).

Annual mean consumption rates remained higher in women, except for two classes higher in men: class B (blood and haematopoietic organs, 13.9 ± 7.6; *P* = .002) and class C (cardiovascular system, 130.1 ± 142.1; *P* = .002). At the ATC2 level, these differences were driven by antithrombotic agents in class B (3.62 ± 2.76 vs 2.63 ± 1.99) and by cardiac therapy and agents acting on the renin–angiotensin system in class C (0.62 ± 0.53 vs 0.53 ± 0.46 and 15.32 ± 14.95 vs 13.55 ± 13.11, respectively; all *P* = .002).

## Discussion

To our knowledge, this study is the first to examine the risk of being exposed to a disruption in drug availability. Using exhaustive national-level data over 10 years, it provides a comprehensive analysis based on drug reimbursement data, encompassing the entire population and including all reported drug shortages.

Our results suggest that age and sex affect the risk of exposure to drug shortages. ≥60-year-old patients and women have a higher risk due to the highest consumption, but <20-year-olds and men show increased exposure in some therapeutic classes, including anti-infectives and agents acting on the renin–angiotensin system, respectively. These disparities suggest differing levels of exposure to drug shortages, likely influenced by distinct healthcare needs and shortage patterns.

A recent study by Orhon *et al.* [25] explored the relationship between drug consumption and reported adverse events in France by comparing pharmacovigilance cases with consumption data. This methodology identified significant correlations between the use of certain drugs and the frequency of reported adverse effects, highlighting the importance of integrating consumption data for a more accurate assessment of drug-related risks.

Our data confirm that drug consumption varies by age and sex, with women showing higher rates than men, consistent with previous studies [26–29]. Although women and the elderly have higher overall drug consumption and specific ATC patterns of consumption, taking into account the specific ATC patterns of shortages, younger people and men could be more impacted by shortages in specific classes.

Our results confirm the increase in reports of unavailability of MTI drugs over time in France (more than 11-fold in 10 years), and particularly those concerning the risk of shortages. Reporting a shortage as soon as a risk of disruption is identified allows for better anticipation of situations that could become critical for patients. However, this proactive approach can also result in informational stress of patients [2], linked to the climate of uncertainty surrounding the potential disruption of treatment. This increase in reporting over time was observed in several therapeutic classes with certain classes being more frequently reported and increasingly affected over time. Results are consistent with the OECD analysis [13], which points out that more than half of all reports were concentrated in three main therapeutic areas: cardiovascular system, nervous system and anti-infective agents.

Over the 10-year study period, regulatory changes were likely to have contributed to modifying the rate of reporting, but these changes were independent of ATC classes or population profile. The mandatory report is defined at the level of the MTI drug with a constant definition over time. If recent regulations, such as mandatory stocks and penalties, may have a positive impact on drug shortage reports in 2024 in France [30], the level remained high and public health impact should be assessed. Comparing different populations’ drug consumption within the same ATC class allows us to identify which groups are most exposed to shortages, while comparisons between classes may be biased by differences in the number of MTI drugs rather than reflecting true shortage rates. One limitation of our study focused on reports of shortages rather than actual treatment interruptions at the patient level. Our analysis therefore reflects an estimation of exposure risk, derived from the combination of regulatory declarations and drug consumption data, and does not directly capture disruptions in patient access or clinical consequences. Our study does not allow us to determine with multivariate analyses whether this risk is specifically related to shortage-prone drugs and the clinical consequences. Reports trigger measures aimed at mitigating shortages, without information on the impact on patients [13]. In its report [30], the French Department of Research, Studies, Evaluation and Statistics emphasizes that a shortage, or the risk of a shortage, did not lead to a collapse in sales at the national level and over a year. This highlights an important distinction between a regulatory drug shortage and a true interruption in patient-level drug supply, which should be considered when interpreting exposure risk estimates. Indeed, it is difficult to determine the extent to which shortage reports have had negative effects on patients’ health. Several therapeutic classes are well documented in the literature as being particularly prone to shortages in hospital settings [31, 32], with significant implications for patient management and clinical outcomes. An American survey [33] has highlighted the fact that the feelings and experiences of patients affected by drug shortages vary according to their sociodemographic characteristics, particularly with regard to age. Future prospective, patient-centered research should aim to quantify the clinical consequences of shortages for inpatients and outpatients [15].

Regarding the data, reimbursement records cover only drugs dispensed in community pharmacies and exclude hospital-only drugs, which represent 4.78% of marketed presentations. Reports, however, cover only MTIs which are particularly relevant for assessing the impact of drug shortages.

Our analysis focuses on the number of reimbursed drug packages, in addition to the number of consumers, to capture the overall volume of drug consumption. This approach highlights the contribution of chronic treatments reflecting longer treatment duration and also considers prescribed dosage, both of which influence the total number of packages consumed over the year. In prospective studies, examining each individual drug specialty in real time would allow a more precise assessment of actual shortages and their patient impact, beyond exposure estimates alone. When analysing drug consumption in terms of the number of reimbursed packages concerned by shortage reports per 1000, these trends remained largely consistent, reinforcing the observed disparities. For the under-20s in particular, the significant difference observed within anti-infective agents is explained by vaccines. However, some differences emerged, notably for respiratory and sensory organ drugs in younger patients and genitourinary drugs in older adults, where initial differences were no longer significant. Similarly, sex-based differences persisted, except for blood and cardiovascular medications, for which the trend was reversed, which remained more frequently consumed by men. Our findings are in line with an Italian study [28], which reported a higher prevalence of drug use and prescriptions among women, while men had longer treatment durations.

Age categories employed provided limited granularity, especially for extreme ages, such as elderly and paediatric populations. These vulnerable populations may be more affected in a shortage context and particularly at risk, as they face greater challenges in adapting to alternative treatments [34, 35]. Recent studies [26, 36, 37] have confirmed that prescribing patterns vary within these age extremes, highlighting the need for further research with more precise population characterization, age stratifications, and clinical factors to better understand their specific impact.

## Conclusion

Our exploratory study indicates that a population-level approach is essential to understand how shortages affect different groups and exacerbate health inequalities. By prioritizing these assessments, future research can support better decision-making and strengthen health systems to protect patient well-being during supply disruptions.

## Supplementary Material

ckag045_Supplementary_Data

## Data Availability

All data concerning drug shortage reports are routinely collected (https://ansm.sante.fr/vos-demarches/industriel/declarer-une-rupture-de-stock-mitm) and contain no information about specific people. Pharmacy drug reimbursement data were extracted and aggregated from the exhaustive French National Health Data System, via the open-access database, Open Medic: https://www.assurance-maladie.ameli.fr/etudes-et-donnees/open-medic-depenses-beneficiaires-medicaments. French demographic data were obtained from the French National Institute of Statistics and Economic Studies (INSEE): https://www.insee.fr/fr/statistiques/series/103088458? INDICATEUR=2771084
