# Supplementary material for: Age and sex disparities in drug shortage impacts: a 10-year nationwide study in France
Source: Eur J Public Health. 2026 Mar 28;36(2):ckag045. doi: 10.1093/eurpub/ckag045 (PMC13032890; doi:10.1093/eurpub/ckag045)
Supplement: ckag045_Supplementary_Data [file ckag045_supplementary_data.zip › ejph-2025-07-om-0593-File006.docx]

**Table S1: Mean annual consumer rate per capita (10-year average) by therapeutic class according to age group or sex**

| **ATC 1 class** | | **Consumer rate** | | | | |  | | | |
| --- | --- | --- | --- | --- | --- | --- | --- | --- | --- | --- |
|  |  | **Age group** | | | | |  | **Sex** | | |
|  |  | **<20 years** |  | **20-59 years** |  | **≥60 years** |  | **Men** |  | **Women** |
|  |  | Mean (SD) |  | Mean (SD) |  | Mean (SD) |  | Mean (SD) |  | Mean (SD) |
| **A** | Digestive system and metabolism | 0.50 (0.02) |  | 0.53 (0.01) |  | 0.79 (0.01) |  | 0.52 (0.01) |  | 0.66 (0.01) |
| **B** | Blood and haematopoietic organs | 0.06 (0.01) |  | 0.15 (0.01) |  | 0.41 (0.00) |  | 0.17 (0.01) |  | 0.22 (0.01) |
| **C** | Cardiovascular system | 0.01 (0.00) |  | 0.14 (0.00) |  | 0.67 (0.02) |  | 0.24 (0.00) |  | 0.25 (0.00) |
| **D** | Dermatology | 0.32 (0.03) |  | 0.33 (0.02) |  | 0.44 (0.02) |  | 0.32 (0.02) |  | 0.39 (0.02) |
| **G** | Genitourinary system and sex hormones | 0.06 (0.00) |  | 0.21 (0.01) |  | 0.19 (0.01) |  | 0.07 (0.00) |  | 0.26 (0.02) |
| **H** | Systemic hormones, excluding sex hormones and insulins | 0.21 (0.03) |  | 0.23 (0.02) |  | 0.32 (0.02) |  | 0.20 (0.02) |  | 0.30 (0.03) |
| **J** | Anti-infective agents (systemic use) | 0.56 (0.04) |  | 0.46 (0.02) |  | 0.69 (0.01) |  | 0.50 (0.02) |  | 0.59 (0.02) |
| **L** | Antineoplastic and immunomodulating agents | 0.00 (0.00) |  | 0.02 (0.00) |  | 0.05 (0.00) |  | 0.02 (0.00) |  | 0.02 (0.00) |
| **M** | Musculoskeletal system | 0.26 (0.07) |  | 0.43 (0.05) |  | 0.47 (0.04) |  | 0.36 (0.05) |  | 0.44 (0.05) |
| **N** | Nervous system | 0.67 (0.04) |  | 0.67 (0.01) |  | 0.78 (0.02) |  | 0.65 (0.02) |  | 0.75 (0.02) |
| **P** | Parasiticides, insecticides and repellents | 0.04 (0.01) |  | 0.04 (0.00) |  | 0.03 (0.00) |  | 0.03 (0.00) |  | 0.05 (0.00) |
| **R** | Respiratory system | 0.49 (0.05) |  | 0.39 (0.03) |  | 0.42 (0.04) |  | 0.39 (0.03) |  | 0.45 (0.04) |
| **S** | Sensory organs | 0.21 (0.02) |  | 0.17 (0.01) |  | 0.33 (0.01) |  | 0.19 (0.01) |  | 0.24 (0.01) |
| **V** | Miscellaneous | 0.01 (0.00) |  | 0.08 (0.01) |  | 0.15 (0.01) |  | 0.07 (0.01) |  | 0.09 (0.01) |
