# Supplementary material for: Age and sex disparities in drug shortage impacts: a 10-year nationwide study in France
Source: Eur J Public Health. 2026 Mar 28;36(2):ckag045. doi: 10.1093/eurpub/ckag045 (PMC13032890; doi:10.1093/eurpub/ckag045)
Supplement: ckag045_Supplementary_Data [file ckag045_supplementary_data.zip › ejph-2025-07-om-0593-File007.docx]

**Table S2: Mean annual consumption rate per capita (10-year average) by therapeutic class according to age group or sex**

| **ATC 1 class** | | **Consumption rate** | | | | |  | | | |
| --- | --- | --- | --- | --- | --- | --- | --- | --- | --- | --- |
|  |  | **Age group** | | | | |  | **Sex** | | |
|  |  | **<20 years** |  | **20-59 years** |  | **≥60 years** |  | **Men** |  | **Women** |
|  |  | Mean (SD) |  | Mean (SD) |  | Mean (SD) |  | Mean (SD) |  | Mean (SD) |
| **A** | Digestive system and metabolism | 1.68 (0.13) |  | 3.87 (0.09) |  | 13.32 (0.30) |  | 4.87 (0.18) |  | 6.65 (0.19) |
| **B** | Blood and haematopoietic organs | 0.18 (0.02) |  | 0.90 (0.04) |  | 5.14 (0.06) |  | 1.84 (0.08) |  | 1.80 (0.08) |
| **C** | Cardiovascular system | 0.02 (0.00) |  | 1.33 (0.08) |  | 11.70 (0.96) |  | 3.77 (0.12) |  | 3.61 (0.21) |
| **D** | Dermatology | 0.80 (0.09) |  | 1.11 (0.07) |  | 1.91 (0.10) |  | 1.13 (0.05) |  | 1.35 (0.09) |
| **G** | Genitourinary system and sex hormones | 0.20 (0.01) |  | 1.01 (0.11) |  | 1.59 (0.07) |  | 0.67 (0.03) |  | 1.24 (0.14) |
| **H** | Systemic hormones, excluding sex hormones and insulins | 0.38 (0.06) |  | 0.77 (0.11) |  | 1.96 (0.26) |  | 0.62 (0.06) |  | 1.32 (0.19) |
| **J** | Anti-infective agents (systemic use) | 2.16 (0.24) |  | 1.89 (0.16) |  | 3.02 (0.18) |  | 2.01 (0.15) |  | 2.47 (0.18) |
| **L** | Antineoplastic and immunomodulating agents | 0.02 (0.00) |  | 0.21 (0.02) |  | 0.53 (0.04) |  | 0.21 (0.02) |  | 0.29 (0.03) |
| **M** | Musculoskeletal system | 0.47 (0.15) |  | 1.44 (0.27) |  | 2.63 (0.54) |  | 1.38 (0.24) |  | 1.64 (0.34) |
| **N** | Nervous system | 3.03 (0.26) |  | 10.76 (0.15) |  | 25.85 (1.47) |  | 10.56 (0.18) |  | 14.88 (0.41) |
| **P** | Parasiticides, insecticides and repellents | 0.08 (0.01) |  | 0.11 (0.01) |  | 0.08 (0.01) |  | 0.07 (0.01) |  | 0.11 (0.02) |
| **R** | Respiratory system | 1.85 (0.24) |  | 1.83 (0.08) |  | 3.13 (0.13) |  | 2.02 (0.12) |  | 2.32 (0.12) |
| **S** | Sensory organs | 0.43 (0.05) |  | 0.61 (0.02) |  | 2.90 (0.07) |  | 0.97 (0.03) |  | 1.33 (0.03) |
| **V** | Miscellaneous | 0.03 (0.02) |  | 0.12 (0.03) |  | 0.29 (0.10) |  | 0.14 (0.02) |  | 0.15 (0.07) |
